# Whole lung tissue is the preferred sampling method for amplicon-based characterization of murine lung microbiota

**DOI:** 10.1186/s40168-021-01055-4

**Published:** 2021-05-05

**Authors:** Jennifer M. Baker, Kevin J. Hinkle, Roderick A. McDonald, Christopher A. Brown, Nicole R. Falkowski, Gary B. Huffnagle, Robert P. Dickson

**Affiliations:** 1 Department of Microbiology and Immunology, University of Michigan Medical School, Ann Arbor, MI 48109 USA; 2 Division of Pulmonary and Critical Care Medicine, Department of Internal Medicine, University of Michigan Health System, 6220 MSRB III/SPC 5642, 1150 W. Medical Center Dr, Ann Arbor, MI 48109-5642 USA; 3 Department of Molecular, Cellular, & Developmental Biology, University of Michigan, Ann Arbor, MI 48109 USA; 4 Mary H. Weiser Food Allergy Center, University of Michigan Medical School, Ann Arbor, MI 48109 USA; 5 Michigan Center for Integrative Research in Critical Care, Ann Arbor, MI USA

**Keywords:** Lung microbiome, 16S rRNA gene amplicon sequencing, Bronchoalveolar lavage, Whole lung tissue

## Abstract

**Background:**

Low-biomass microbiome studies (such as those of the lungs, placenta, and skin) are vulnerable to contamination and sequencing stochasticity, which obscure legitimate microbial signal. While human lung microbiome studies have rigorously identified sampling strategies that reliably capture microbial signal from these low-biomass microbial communities, the optimal sampling strategy for characterizing murine lung microbiota has not been empirically determined. Performing accurate, reliable characterization of murine lung microbiota and distinguishing true microbial signal from noise in these samples will be critical for further mechanistic microbiome studies in mice.

**Results:**

Using an analytic approach grounded in microbial ecology, we compared bacterial DNA from the lungs of healthy adult mice collected via two common sampling approaches: homogenized whole lung tissue and bronchoalveolar lavage (BAL) fluid. We quantified bacterial DNA using droplet digital PCR, characterized bacterial communities using 16S rRNA gene sequencing, and systematically assessed the quantity and identity of bacterial DNA in both specimen types. We compared bacteria detected in lung specimens to each other and to potential source communities: negative (background) control specimens and paired oral samples. By all measures, whole lung tissue in mice contained greater bacterial signal and less evidence of contamination than did BAL fluid. Relative to BAL fluid, whole lung tissue exhibited a greater quantity of bacterial DNA, distinct community composition, decreased sample-to-sample variation, and greater biological plausibility when compared to potential source communities. In contrast, bacteria detected in BAL fluid were minimally different from those of procedural, reagent, and sequencing controls.

**Conclusions:**

An ecology-based analytical approach discriminates signal from noise in this low-biomass microbiome study and identifies whole lung tissue as the preferred specimen type for murine lung microbiome studies. Sequencing, analysis, and reporting of potential source communities, including negative control specimens and contiguous biological sites, are crucial for biological interpretation of low-biomass microbiome studies, independent of specimen type.

Video abstract

**Supplementary Information:**

The online version contains supplementary material available at 10.1186/s40168-021-01055-4.

## Background

Though the development of next-generation sequencing has led to heightened interest in the study of microbial communities across biological contexts, the study of low-biomass microbiomes is particularly challenging and requires the development of new methodological approaches. Low-biomass samples—samples with low densities of bacterial cells and therefore low quantities of bacterial DNA—are susceptible to contamination with background-derived signal, which affects the taxonomic composition of low-biomass samples [1, 2]. This challenge of background DNA, contamination, and sequencing stochasticity (here collectively referred to as “noise”) intermingled with legitimate bacterial signal originating from a biological specimen (here referred to as “signal”) makes it challenging to decipher biological meaning from sequencing data [3]. These methodological challenges exist in all fields that study low-biomass microbial communities across environmental, industrial, and biomedical contexts.

Low-biomass microbiome fields have had variable success in overcoming these methodological challenges. Whereas early findings related to the purported placenta microbiome have subsequently been attributed to contamination [4, 5], the lung microbiome field has flourished with robust, validated findings: lung microbiota are detectable in health [6–13], correlated with lung immunity both in health [7, 8, 14, 15] and disease [16–18], correlated with disease severity and predictive of response to therapy [19–22], and prognostic of clinical outcomes in multiple conditions [23–30]. The lung microbiome field addressed the challenge of low-biomass microbiome sampling in humans by systematically defining methods that collect representative populations of lung microbiota to maximize bacterial DNA content and minimize vulnerability to background contamination [9–12]. As a result, empirically validated sampling approaches such as bronchoalveolar lavage (BAL) fluid, which (in humans) samples a large surface area and yields high sample volumes, have been successfully implemented in lung microbiome studies [31].

Yet despite their routine use in human lung microbiome studies, these sampling methods are not easily adapted for sampling lung microbiota in murine models, which will be critical to understand the mechanisms that govern the relationship between respiratory tract microbiota and pulmonary disease. Anatomic considerations make the application of sequencing-based techniques to murine lung microbiome studies particularly challenging, as collection of BAL fluid is severely limited by the small (~1 mL) volume of the murine lung and terminal nature of the procedure in mice [32]. In contrast, analysis of homogenized lung tissue is more feasible in mice than humans and represents a viable option for maximizing the bacterial DNA content in murine lung samples [33]. Thus, the ability to effectively sample low-biomass microbial communities is inherently context-dependent and will require new solutions adapted to the particular context of each study.

**Fig. 1:**
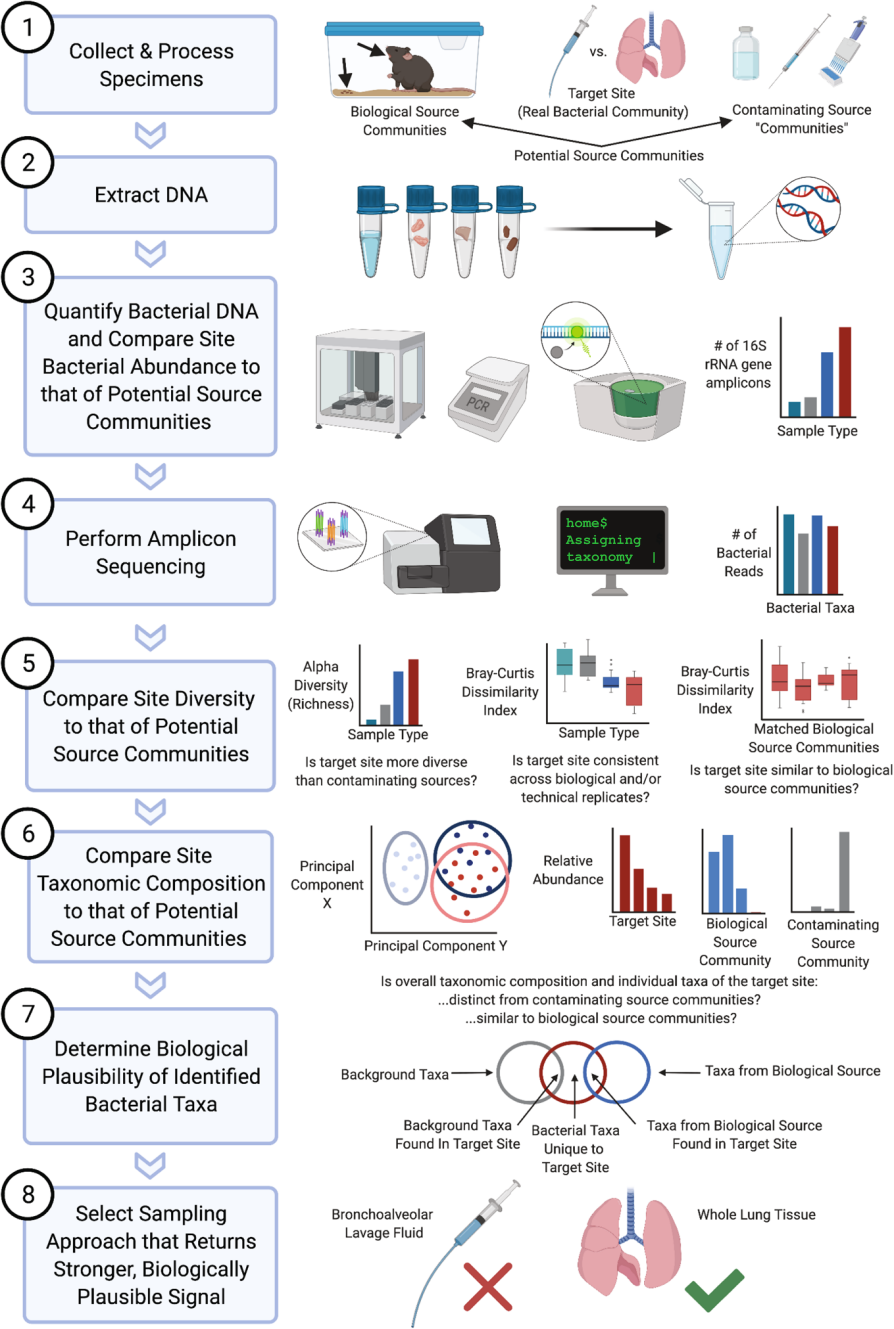
An ecology-based experimental and analytic approach can distinguish bacterial signal from noise in low-biomass microbiome studies. Graphical and conceptual outline of an experimental and analytic approach to low-biomass microbiome studies. This approach was applied to murine lung microbiome sampling optimization in this study and may be useful in other low-biomass microbiome studies across biological contexts

We therefore designed an empirical approach to compare microbial signal detected in two distinct sample types collected from the same ecological site (murine lungs) with the following goal: to determine the sampling method that is best suited for the characterization of the murine lung microbiome. To accomplish this goal, we quantified and sequenced the bacterial DNA present in BAL fluid and whole lung tissue from otherwise genetically—and environmentally—identical healthy mice and compared them using a microbial ecology-based analytic approach (Fig. 1).

## Methods

### Mice

Eight-week-old female C57BL/6 mice (*n* = 20) were purchased from Jackson Laboratories and housed under specific pathogen–free conditions. Mice were housed in five-animal cages in a common animal housing room and did not receive independent ventilation. Mice were allowed to acclimate for 1 week before harvest at 9 weeks of age. To avoid batch effect, mice were randomly assigned to specimen type (BAL fluid or whole lung tissue) and evenly sampled across cages. Animal experimentation was performed in compliance with the ARRIVE Guidelines [34, 35].

### Tissue collection and processing

On the day of harvest, mice were randomized to either the whole lung tissue or BAL fluid groups (Fig. 1, step 1). To account for cage effect during downstream analysis, two or three mice per five-mouse cage were randomly assigned to each sampling group, for a total of 10 mice from each of the four cages in the whole lung tissue and BAL fluid groups. Mice were sacrificed via CO_2_ asphyxiation, and organs were harvested in the following order: tongue, whole lung tissue or BAL fluid, and cecum. A summary of all tissue samples and controls used in this study is provided in Supplementary Table 1. All tissue samples were collected using sterile technique, and instruments were rinsed with ethanol and flamed between each organ. Tongue (*n*=20) and cecum (*n*=20) samples, which were collected to serve as low- and high-biomass positive controls, respectively, were immediately snap-frozen using liquid nitrogen and stored at −80°C until DNA isolation.

Both types of lung samples collected for this study were harvested and processed according to previously published protocols [6, 36], described in brief below. Murine whole lung tissue (*n*=10) was excised, placed in tubes containing 1 mL sterile water, and mechanically homogenized using a Tissue-Tearor (Biospec Products, Bartlesville, OK). The tissue homogenizer was cleaned and rinsed in ethanol and water between each tissue sample. Water control specimens from homogenization (*n*=2) rinsed with clean instruments were included as procedural controls for whole lung tissue. Lung homogenate was centrifuged at 30,000 rpm, supernatant was removed, and the cellular pellet was snap-frozen with liquid nitrogen and stored at −80°C until DNA isolation.

BAL fluid (*n*=10) was collected by (1) sterile dissection to expose and make a small incision in the trachea, (2) insertion of a piece of sterile tubing (BD Intramedic polyethylene tubing, 0.58-mm internal diameter, catalog no. 427410) and connected sterile syringe needle (BD PrecisionGlide^TM^ 23 gauge needle, catalog no. 305145) into the incision, (3) tightening of a piece of sterile surgical thread around the intubated trachea to create an air-tight seal, and (4) two rounds of instillation and retrieval of 1 mL of sterile phosphate-buffered saline (PBS) into the lungs using a sterile syringe (BD 1 mL sterile syringe, catalog no. 309659). Each tubing-needle-syringe setup was rinsed thoroughly with sterile PBS between the collection of each sample. Sterile PBS (*n*=2) used for lavage and PBS rinses (*n*=4) of the syringe, needle, and tubing (pre- and post-lavage) were collected as procedural controls. BAL fluid was prepared by pooling the two serial lavages from each mouse, yielding up to 2 mL total BAL fluid per mouse. Pooled BAL fluid was centrifuged at 30,000 rpm for 30 min, supernatant was removed, and the cellular pellet was snap-frozen with liquid nitrogen and stored at −80°C until DNA isolation.

### DNA extraction, quantification, and 16S rRNA gene sequencing

DNA was extracted, amplified, and sequenced according to previously published protocols [37, 38] (Fig. 1, steps 2 and 4). DNA isolation was performed with a single kit according to a modified protocol previously demonstrated to isolate bacterial DNA [37]. Briefly, genomic DNA was extracted from mouse tissue samples using a DNeasy Blood & Tissue kit (Qiagen, Hilden, Germany, catalog no. 69506) and homogenized in PowerBead Tubes (Qiagen, Hilden, Germany, catalog no. 13123-50). To detect contamination introduced by the DNA isolation kit, elution (AE) buffer (*n*=6) and specimen-free DNA isolations using empty bead tubes (*n*=6) were collected and sequenced as negative controls. Samples were processed in a randomized order to reduce false pattern formation due to reagent contamination [1].

### Bacterial DNA quantification

Bacterial DNA in lung specimens and negative controls was quantified with a QX200 ddPCR system (Bio-Rad, Hercules, CA) according to a previously published protocol [39]. Sampling and DNA isolation controls and sterile PCR-grade water used for sample dilution as a no template control (*n*=4) were run alongside lung specimens. All lung specimens and negative controls were run with two technical replicates. Droplets were generated using an automated droplet generator (Bio-Rad, catalog no. 1864101). PCR amplification was performed with the Bio-Rad C1000 Touch Thermal Cycler (catalog no. 1851197). Primers were 5′- GCAGGCCTAACACATGCAAGTC-3′ (63F) and 5′- CTGCTGCCTCCCGTAGGAGT-3′ (355R). The cycling protocol was 1 cycle at 95°C for 5 min, 40 cycles at 95°C for 15 s and 60°C for 1 min, 1 cycle at 4°C for 5 min, and 1 cycle at 90°C for 5 min, with all steps at a ramp rate of 2°C/s. Droplets were detected using the automated droplet reader (Bio-Rad, catalog no. 1864003), quantified using Quantasoft^TM^ Analysis Pro (version 1.0.596), and imported to R (version 4.0.2) for visualization and statistical analysis.

### 16S rRNA gene sequencing

The V4 region of the 16S rRNA gene was amplified using published primers [40] and the dual-indexing sequencing strategy developed by the laboratory of Patrick D. Schloss [38], according to the manufacturer’s instructions with modifications found in the Schloss standard operating procedures (SOP) [41] as published previously [42, 43]. For primary PCR, each 20 μL PCR reaction contained the following: 5 μL of a 4 μM equimolar primer set, 2 μL 10X AccuPrime PCR Buffer II (Life Technologies, catalog no. 12346094), 9.85 μL sterile PCR-grade water, 0.15 μL Accuprime High Fidelity Taq Polymerase (Life Technologies catalog no. 12346094), and 3 μL of template DNA. PCR cycling conditions were 95°C for 2 min, followed by 20 cycles of touchdown PCR (95°C 20 s, 60°C 15 s and decreasing 0.3° each cycle, 72°C 5 min), then 20 cycles of standard PCR (95°C for 20 s, 55°C for 15 s, and 72°C for 5 min), and finished with 72°C for 10 min. PCR products were visualized using an E-Gel 96 with SYBR Safe DNA Gel Stain, 2% (Life Technologies catalog no. G7208-02). Samples that did not amplify during the first round of PCR were reamplified during a single round of troubleshooting using the same PCR cycling protocol and reaction composition as described above, except for the following modifications: increasing template DNA volume from 3 to 5 μL and decreasing the sterile PCR-grade water volume by 2 μL to yield a total reaction volume of 20 μL.

After confirming successful amplification of all samples, libraries were normalized using SequalPrep Normalization Plate Kit (Life Technologies, catalog no. A10510-01) following the manufacturer’s protocol for sequential elution. The concentration of the pooled samples was determined using Kapa Biosystems Library Quantification kit for Illumina platforms (Kapa Biosystems, catalog no. KK4824), and amplicon size was determined using the Agilent Bioanalyzer High Sensitivity DNA analysis kit (catalog no. 5067-4626). Libraries were prepared according to Illumina’s “Preparing Libraries for Sequencing on the MiSeq” protocol for 2 nM libraries (part no. 15039740 Rev. D). The final library consisted of equimolar amounts from each of the plates normalized to the pooled plate at the lowest concentration. The final load concentration was 5 pM, spiked with PhiX at 15% to add diversity. Sequencing reagents were prepared according to the Schloss SOP, and custom read 1, read 2, and index primers were added to the reagent cartridge. Amplicons were sequenced using the Illumina MiSeq platform (San Diego, CA) using a MiSeq Reagent Kit V2 (Illumina, catalog no. MS102-2003) for 500 cycles. Sterile water (*n*=8) and empty wells (*n*=28) were sequenced as negative controls, and a synthetic community (*n*=4; ZymoBIOMICS Microbial Community DNA Standard, Zymo Research catalog no. D6306) was sequenced as a positive control. FASTQ files were generated with paired end reads and retained for further analysis.

### Adequacy of sequencing

The full dataset obtained from the sequencing run included 5,560,120 total reads, with a mean ± SD of 46,334 ± 63,233 reads per specimen and a range of 53–287,832 reads per specimen. The dataset post-*mothur* processing included 2,062,759 identified bacterial reads, with a mean ± SD of 17,190 ± 23,515 reads per specimen and a range of 12–113,592 reads per specimen. One whole lung tissue sample, one isolation control, one elution buffer control, two water controls, and four empty well controls were identified as having an insufficient number of sequencing reads (< 150 reads) and were excluded from further analysis. All other tissue and control samples met the minimum requirement for number of sequencing reads (≥ 150 reads) and were retained for further analysis (Supplementary Figure 1). No major differences between the conclusions drawn from hypothesis tests conducted with the full sequencing dataset compared to the trimmed, quality-checked sequencing dataset were observed (Supplementary Table 2).

### Data analysis

16S rRNA gene sequencing data were processed using mothur (v. 1.43.0) according to the Standard Operating Procedure for MiSeq sequence data using a minimum sequence length of 250 base pairs [41, 44]. A shared community file and a genus-level phylotyping file were generated using operational taxonomic units (OTUs) binned at 97% identity, using SILVA (v. 132) for sequence alignment (silva.nr_v132.regionV4.align). OTU numbers were arbitrarily assigned in the binning process and are referred to throughout the manuscript in association with their most specified level of taxonomy (typically genus or family). OTUs were classified using the mothur implementation of the Ribosomal Database Project (RDP) classifier and RDP taxonomy training set 16 (trainset16_022016.rdp.fasta, trainset16_022016.rdp.tax), available on the mothur website [41]. After data processing with mothur, shared community (OTU) and taxonomy files were imported to R for trimming and quality checks. OTUs that composed greater than 0.1% of reads in all samples were retained in the trimmed dataset for further analysis. One sample (WVB_Lung_L3) yielded less than 150 reads and was removed from the quality-checked dataset; all other experimental and control samples were retained in the quality-checked dataset, which was used for the main analysis.

Microbial community analysis of the quality-checked dataset was performed in R (version 4.0.2) [45] and relied primarily on the tidyverse (v. 1.3.0) [46], ggplot2 (v. 3.3.0) [47], vegan (v. 2.5-6) [48], and cbmbtools (v. 0.0.09025) [49] packages. For relative abundance, samples were normalized to the percent of total reads, and analysis was restricted to OTUs that were present at greater than 0.1% of the sample population. No OTUs were excluded from the dataset to account for background contamination. Diversity comparisons were performed by calculating community richness rarefied to 100 reads per sample, Shannon diversity index, and the Bray-Curtis dissimilarity index. Ordinations were performed using principal component analysis on Hellinger-transformed OTU count tables generated using Euclidean distances [50].

Overall significance was determined as appropriate by the Kruskal-Wallis test and by permutational multivariate analysis of variance (PERMANOVA) with 10,000 permutations using Hellinger-transformed OTU count tables and Euclidean distances with the adonis function in the R package vegan. Pairwise significance was determined as appropriate by the Wilcoxon test with the Benjamini-Hochberg correction for multiple comparisons, Tukey’s honest significant difference (HSD) test, two-sample independent Mann-Whitney *U* test, and two-group PERMANOVA as described for overall significance testing. All statistical tests used *p*=0.05 as a threshold for significance.

## Results

### Murine whole lung tissue contains more bacterial DNA than BAL fluid and negative controls

**Fig. 2:**
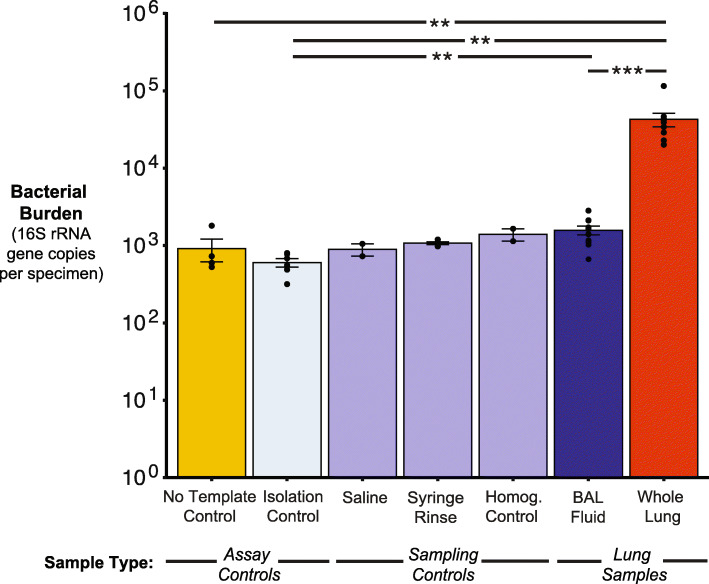
Murine whole lung tissue contains increased bacterial burden relative to BAL fluid and negative controls. Whole lung tissue contains more copies of the bacterial 16S rRNA gene per mL of DNA isolated from lung or control specimens as quantified by ddPCR. Mean ± SEM and individual data points (representing the average of technical duplicates) are shown. Overall significance was determined by the Kruskal-Wallis test (*p* = 0.00005). Pairwise significance was determined by the pairwise Wilcoxon test and corrected for multiple comparisons using the Benjamini-Hochberg method (pairwise comparisons including whole lung or BAL fluid that are not shown were not significant). Significance key: *p ≤ 0.05; **p ≤ 0.01; ***p ≤ 0.001; ****p ≤ 0.0001

Obtaining quality sequencing data depends on the presence of sufficient bacterial DNA in the samples to be analyzed. Therefore, we first compared the quantity of bacterial DNA in whole lung tissue and BAL fluid obtained from healthy C57BL/6 mice (Fig. 1, step 3). We hypothesized that whole lung tissue contains more bacterial DNA compared to BAL fluid. To test this hypothesis, we determined the number of 16S rRNA gene copies present in DNA isolated from whole lung tissue, BAL fluid, and negative control specimens using droplet digital PCR (ddPCR). As seen in Fig. 2, BAL fluid and whole lung tissue both contained a significantly greater quantity of bacterial DNA than the isolation control (*p*=0.008 and 0.003, respectively). In contrast, BAL fluid did not contain more bacterial DNA than sampling controls or no template controls (*p*>0.05). Whole lung tissue contained significantly more bacterial DNA than all other groups, including all negative controls (*p*=0.00005). Whole lung tissue contained 27-fold more 16S rRNA gene copies than BAL fluid (42,740 vs. 1578 mean copies/specimen, respectively; *p*=0.0002). We thus concluded that murine whole lung tissue contains a greater quantity of bacterial DNA than does BAL fluid.

Having confirmed the presence of detectable bacterial DNA in whole lung tissue and BAL fluid, we proceeded with 16S rRNA gene sequencing according to a standard low-biomass protocol. Along with whole lung tissue and BAL fluid, we sequenced a variety of controls, including cecum as a high-biomass positive control, tongue as a low-biomass positive control and potential source community of the lower respiratory tract, a synthetic mock community as a positive sequencing control, and negative controls for each stage of specimen processing, including sampling, DNA isolation, and sequencing controls. Despite the increased amount of bacterial DNA in whole lung tissue, BAL fluid returned a greater number of reads than whole lung tissue. These results are consistent with the compositional, rather than quantitative, nature of amplicon-based sequencing, and are likely attributable to the high host-to-bacteria ratio in whole lung tissue (Supplementary Figure 1). With the exception of one whole lung tissue sample and five negative controls, all other specimens returned an adequate number of reads for further analysis. More details regarding adequacy of sequencing are provided in the “Methods” section.

### Murine whole lung tissue has increased alpha diversity and decreased sample-to-sample variation relative to BAL fluid and negative controls

**Fig. 3:**
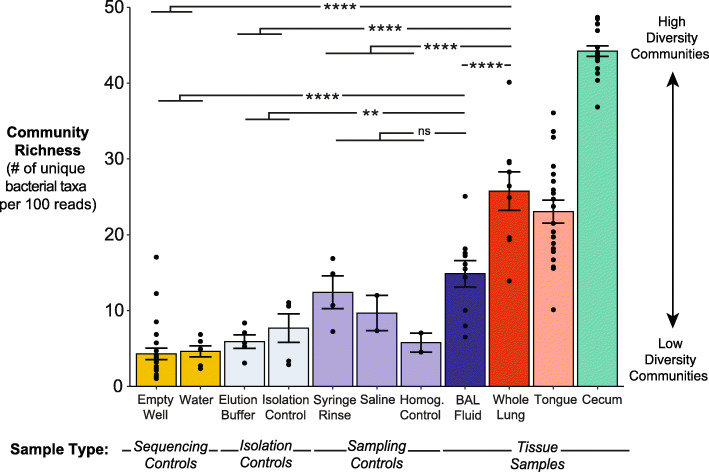
Bacterial communities in murine whole lung tissue have increased alpha diversity relative to BAL fluid and negative controls. **a** Whole lung tissue contains a greater number of unique bacterial taxa than BAL fluid and negative controls. Richness of the bacterial community in each tissue or control specimen was determined by clustering reads with species-level similarity (≥ 97% sequence identity) into operational taxonomic units (OTUs) and calculating the number of unique OTUs within each specimen, normalized to 100 reads per specimen to account for variation in sequencing depth. Mean ± SEM and individual data points are shown. Pairwise significance was determined by comparing whole lung tissue and BAL fluid to pooled sampling, isolation, and sequencing controls (respectively, as shown) using Tukey’s Honest Significant Difference (HSD) test. Significance key: ns *p* > 0.05; *p ≤ 0.05; **p ≤ 0.01; ***p ≤ 0.001; ****p ≤ 0.0001

We next determined if the alpha (within-sample) diversity also differed across sampling approaches (Fig. 1, step 5). We hypothesized that the increased quantity of bacterial DNA in whole lung tissue would yield greater diversity of bacterial taxa in whole lung tissue compared to BAL fluid. To test this hypothesis, we calculated community richness as measured by the number of unique operational taxonomic units (OTUs) per 100 reads present in each specimen and negative control. As predicted, whole lung tissue had greater community richness than BAL fluid (*p*=0.00002) and sampling, isolation, and sequencing controls (*p*<0.0001 for all comparisons) (Fig. 3). In contrast, whole lung and BAL specimens did not significantly differ in Shannon diversity index, which reflects both community richness and evenness (*p*>0.05; Supplementary Figure 2). We therefore concluded that alpha diversity differs across sampling approaches, with greater alpha diversity in whole lung tissue driven by the detection of greater numbers of unique OTUs relative to BAL fluid.

**Fig. 4:**
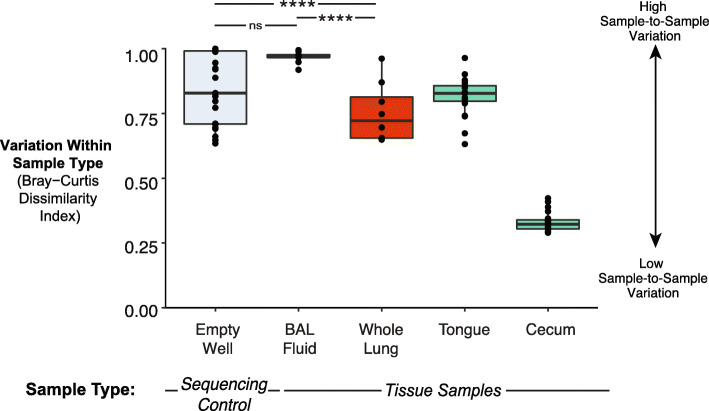
Bacterial communities in murine whole lung tissue show decreased variation among biological replicates compared to those in BAL fluid. Variation among lung bacterial communities of healthy mice from the same shipment was quantified using the Bray-Curtis dissimilarity index. For comparison, Bray-Curtis dissimilarity was also calculated for empty wells as a representative negative control with high variation, cecal communities as a representative body site with low variation, and tongue as a representative seed community for the lower respiratory tract. Median, IQR, and all unique pairwise comparisons (individual data points) are shown. Pairwise significance was determined by pairwise Wilcoxon test and corrected for multiple comparisons using the Benjamini-Hochberg method. Significance key: ns *p* > 0.05; *p ≤ 0.05; **p ≤ 0.01; ***p ≤ 0.001; ****p ≤ 0.0001

Since BAL fluid contained low quantities of bacterial DNA and fewer unique OTUs than whole lung tissue, we suspected that incomplete sampling of the respiratory tract via saline lavage may also result in increased sampling and/or sequencing stochasticity [51], which both lead to decreased specimen-to-specimen reproducibility of cohoused mice. We have previously shown that mice from the same shipment and vendor have similar lung microbiota [6], and thus made this assumption in the following comparison. We hypothesized that whole lung tissue would have decreased sample-to-sample variation relative to BAL fluid, representing greater replicability. To test this hypothesis, we computed the Bray-Curtis dissimilarity index, a beta-diversity metric based on pairwise inter-sample distances between specimens of the same type (i.e., we compared each whole lung tissue specimen to each other whole lung tissue specimen, and likewise for BAL fluid). Whole lung tissue yielded a decrease in average Bray-Curtis dissimilarity index relative to that of BAL fluid and empty well controls (*p*<0.0001) (Fig. 4). In contrast, the average Bray-Curtis dissimilarity index for BAL fluid was not significantly different than the highly dissimilar empty well controls (*p*=0.3). The high dissimilarity among BAL fluid samples is likely attributable to the low amount of bacterial DNA present in this sample type, as previous work from our group showed an increase in sequencing stochasticity as bacterial biomass of the sample decreases [51]. Notably, the average Bray-Curtis dissimilarity index for whole lung tissue still showed a large amount of variation (median = 0.72). Since technical replicates were not sequenced in this study, the degree to which sequencing stochasticity contributes to the high amount of variation observed among lung microbiota from either sample type remains unknown. However, this variation observed in whole lung tissue is at least partly attributable to the sampling of mice from four different cages and the innate microbiological variation that occurs when sampling a single timepoint in the lung, which experiences rapid turnover of microbiota due to constant immune surveillance [14]. By comparison, the average Bray-Curtis dissimilarity index for whole lung tissue was lower than that of oral samples, supporting the biological plausibility of the observed variation between whole lung tissue samples. Taken together, these results suggest that whole lung tissue displays decreased sample-to-sample variation and likely samples the lung microbiome of mice more reproducibly than BAL fluid.

### The taxonomic composition of murine whole lung tissue is similar to its oral microbiome source community and is distinct from negative controls, whereas that of BAL fluid is not distinct from negative controls

**Fig. 5:**
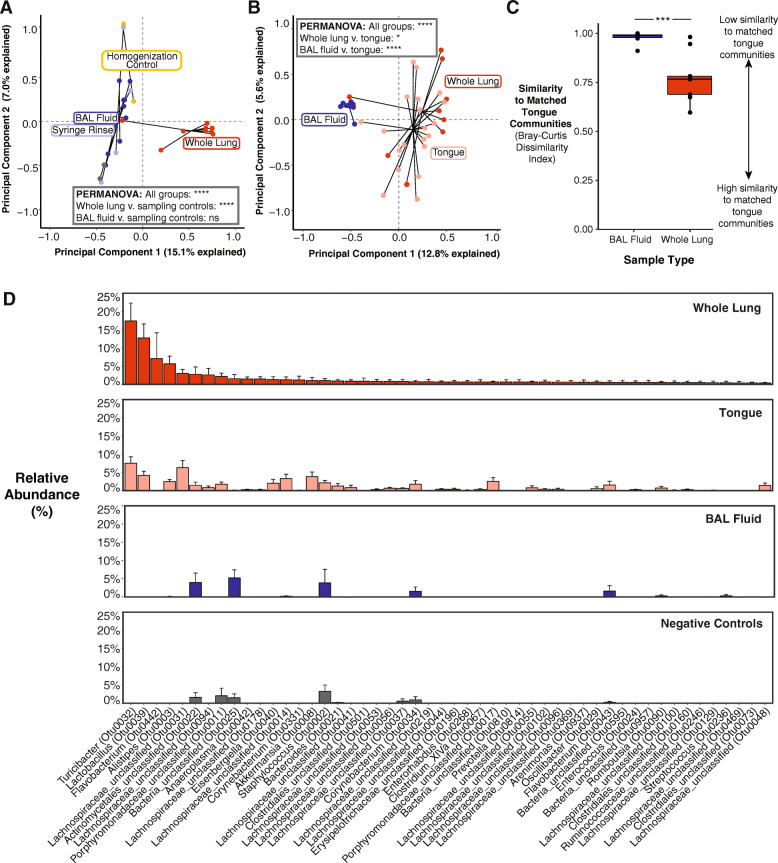
The taxonomic composition of bacterial communities in murine whole lung tissue is distinct from the background-dominant taxonomic composition of BAL fluid and similar to that of the oral microbiome, a biologically plausible source community. **a** Whole lung tissue clusters separately from BAL fluid and sampling controls by principal component analysis of Hellinger-transformed 16S rRNA gene sequencing data. Individual data points represent specimens grouped by sample or control type. **b** Whole lung tissue, but not BAL fluid, clusters near tongue samples by principal component analysis of Hellinger-transformed 16S rRNA gene sequencing data. Individual data points represent specimens grouped by sample type. **c** Bacterial communities in whole lung tissue are more similar to matched (within-mouse) oral communities than BAL fluid. Similarity of lung bacterial communities, grouped by sampling approach, to matched oral communities was quantified using Bray-Curtis dissimilarity index. Median, IQR, and individual data points representing within-mouse comparisons of oral and lung communities are shown. **d** Relative abundance of bacterial taxa in whole lung tissue are similar to that of oral bacterial communities. In contrast, the relative abundance of bacterial taxa in BAL fluid is similar to that of negative controls. Bars are ranked by mean abundance in whole lung tissue and represent mean ± SEM percent relative abundance of the top 50 bacterial taxa (OTUs) in whole lung tissue across sample types. Labels denote genus (or most specific taxonomic level if no genus was assigned) and unique identifier for each OTU. Overall significance was determined by (**a**, **b**) permutational multivariate ANOVA (*p* = 0.00009 for both). Pairwise significance was determined by (**a**, **b**) two-sample PERMANOVA (**a** only: pooled sampling controls were compared to each lung sample type), and **c** two-sample unpaired Mann-Whitney *U* test. Significance key: ns *p* > 0.05; *p ≤ 0.05; **p ≤ 0.01; ***p ≤ 0.001; ****p ≤ 0.0001

Having identified differences in bacterial quantity and diversity across sampling approaches, we next assessed whether the taxonomic composition of whole lung tissue and BAL fluid differed from each other and from negative controls (Fig. 1, step 6). Since whole lung tissue had higher bacterial DNA content and alpha diversity than BAL fluid, we hypothesized that the taxonomic composition of BAL fluid would more closely resemble that of negative control specimens than would whole lung tissue, reflecting background contamination and sequencing noise as predominant sources of taxa in BAL fluid. To test this hypothesis, we used principal component analysis (PCA) to compare the similarity of taxa identified in whole lung tissue, BAL fluid, and negative control specimens. As seen in Fig. 5a, the taxonomic composition of whole lung tissue was distinct from that of BAL fluid (*p*=0.00009) and pooled sampling controls (*p*=0.0004). In contrast, BAL fluid showed prominent overlap with sampling controls and did not differ in overall community composition (*p*=0.46). Similar results were obtained when comparing whole lung tissue and BAL fluid with isolation and sequencing controls (Supplementary Figure 3AB). Overall, these data show that the taxonomic composition of whole lung tissue is distinct from that of BAL fluid and negative controls, whereas BAL fluid is not distinct from most negative controls.

We next assessed the biological plausibility of bacterial taxa by comparing whole lung tissue and BAL fluid communities to their likely source community, the oral microbiome (Fig. 1, step 7). We hypothesized that the taxonomic composition of whole lung tissue would more closely resemble that of the oral microbiome source community than does BAL fluid. Principal component analysis confirmed that tongue and whole lung tissue display similar but statistically different (*p*=0.01) taxonomic compositions, whereas BAL fluid clusters separately both from tongue (*p*=0.00009) and whole lung tissue (Fig. 5b). We confirmed these results by calculating the Bray-Curtis dissimilarity index for matched (i.e., from the same mouse) tongue and lung samples (Fig. 5c). Consistent with the PCA results, whole lung tissue more closely resembled the oral source community than did BAL fluid (*p*=0.0004). Rank abundance analysis revealed that the prominent taxa in whole lung tissue were also common in tongue specimens, whereas taxa in BAL fluid bore little resemblance to oral taxa and instead resembled taxa in negative controls (Fig. 5d). The similarity of taxa in the whole lung and tongue samples and the BAL fluid and negative control samples, respectively, can also be observed when ordering rank abundance plots by the taxa found in the tongue or pooled negative controls (Supplementary Figure 4). Together, these results confirm that the bacterial taxa identified in whole lung tissue are more biologically plausible than those detected in BAL fluid (Fig. 1, step 8).

## Discussion

This study illustrates how an ecology-based analytical approach can interrogate the reality of bacterial signal in low-biomass microbiome studies. Our approach revealed the superiority of murine whole lung tissue relative to BAL fluid in detecting bacterial signal and validates the use of whole lung tissue for lung microbiome studies in mice. The bacterial signal in murine whole lung tissue is stronger than that of BAL fluid by all comparisons: increased quantity of bacterial DNA, greater diversity of bacterial taxa, and taxonomic composition that is reproducible across biological replicates, distinct from negative controls, and more similar to the oral microbiome, a biologically plausible source community (Table 1).

**Table 1:** Comparison of sampling methods for murine lung microbiome studies

		Whole lung tissue	Bronchoalveolar lavage fluid
**Sample description**	Sample content	All lung lobes homogenized in sterile water	Dislodged airway and alveolar contents (microbes, leukocytes, epithelial cells) in sterile saline
	Biological site sampled	Airway and intra-alveolar space, interstitium, and blood (if not perfused)	Airway and intra-alveolar space only
	Bacterial biomass	Low	Low
	Host-to-microbe DNA ratio	High	Low
**Summary of findings** (relative to each other)	Total DNA content	High	Low
	Bacterial DNA content	High	Low
	Variation among biological replicates	Low	High
	Similarity to contaminating source “communities” (negative controls)	Low	High
	Similarity to biological source community (oral microbiome)	High	Low

This study represents the first systematic comparison of sampling methods appropriate for the study of the murine lung microbiome. The lack of empirically validated methods for sampling lung microbiota in mice is particularly concerning in light of the current reproducibility crisis [52] and recent controversial low-biomass studies [4, 5, 53], which highlight the dangers of over-interpreting noisy sequencing data in the absence of rigorous, field-specific standards. A systematic examination of methods for sampling lung microbiota in mice is overdue, especially considering the first report describing the murine lung microbiome was published almost a decade ago [54]. Published murine lung microbiome studies to date have used both whole lung tissue [6, 36, 55–59] as well as BAL fluid [60–62], but no study to date has directly compared sampling approaches. Based on the findings of the current study, we strongly recommend whole lung tissue as a preferred sampling strategy for subsequent murine lung microbiome studies.

While BAL fluid in mice contains weak bacterial signal relative to lung tissue, in humans, the opposite has been observed: human BAL specimens contain consistently stronger bacterial signal than lung tissue acquired via biopsy. This observation is consistent with anatomic and ecologic differences across species. Anatomically, human lungs are much larger than murine lungs, providing increased surface area for sampling (~75 m^2^ vs. 0.008 m^2^) and more airspace (6 L vs. 0.001 L) to accommodate the collection of far larger volumes of BAL fluid [32, 33, 63]. Biopsy specimens of human lungs are typically small in volume and peripheral in anatomic location, meaning they are predominantly composed of interstitium rather than airways and alveolar space (where bacteria are more likely to be found). In contrast, use of whole lung homogenate in mice ensures capture of all bacterial DNA within the entire respiratory tract. Thus, anatomic and ecologic differences between humans and mice necessitate the use of murine-specific sampling approaches and illustrate why a “one-size-fits-all” approach to low-biomass microbiome sampling is unlikely to work: sampling strategies will need to be tailored to their specific environmental and biologic contexts.

Numerous sources of false signal can confound detection of bacterial communities in low-biomass microbiome studies, including contamination (procedural, reagent, and sequencing) and sequencing stochasticity. Salter and colleagues elegantly demonstrated the susceptibility of low-biomass samples to reagent contamination by sequencing serial dilutions of a pure bacterial culture, where increasingly diluted specimens contained increasing abundances of taxa found in the DNA isolation reagents [1]. Other sources of contamination, such as those introduced during specimen collection (e.g., bronchoscope, surgical instruments, collection tubes) or sequencing (e.g., well-to-well contamination or index switching) may also alter the taxonomic composition of low-biomass samples [64, 65]. Additionally, it has recently been demonstrated via the use of sequencing replicates that sequencing stochasticity is itself a major source of variability in microbial signal in low-biomass studies [51]. Given the numerous sources of potential false signal in low-biomass microbiome studies, we do not believe this methodological challenge can be sufficiently addressed with a simple, universal solution (e.g., a single bioinformatic “decontamination” step). Rather, as illustrated in our approach, we believe the reality of microbiologic signal must be assessed within the specific ecologic context from which it is sampled, and anchored in an understanding of microbial ecology.

Several approaches to false signal in low-biomass microbiome studies have been proposed. Strategies used to detect, interpret, and, in some cases, eliminate contamination have included exclusion of taxa detected in negative controls through statistical packages [66, 67] or unbiased subtraction [7], extraction and sequencing technical replicates [51], calculation of abundance ratios [68], correlation analyses [69], hierarchical clustering [70], and building neutral models [71]. In this study, we implement an experimental and analytical approach grounded in principles of microbial ecology to discriminate true microbial signal from background-derived signal. Fundamentally, this approach relies on sampling the low-biomass body site of interest and comparing the size, diversity, and taxonomic composition of the microbial community identified at that low-biomass site to all potential source communities, including background signal derived from procedural, reagent, and sequencing contamination and true microbial signal derived from contiguous body sites. This approach can thus be applied to a single specimen type to discern true bacterial signal from background-derived noise or to compare multiple specimen types to determine the optimal sampling method in the absence of a gold standard. We advocate for this approach to be applied to all low-biomass studies, as the choice of sampling method and resulting sample type should reflect that which yields the strongest biological signal. Of note, this approach does not preclude the use of complementary methods (such as those intended to handle contamination mentioned above), but rather builds a foundation rooted in thorough experimental design, which can then be subjected to further analysis with other bioinformatic tools.

There are several limitations to our study. We selected methods of harvesting BAL fluid and whole lung tissue which have been used by our lab and others successfully, and thus cannot directly speak to other approaches (e.g., use of lung portions or pooled BAL specimens from multiple mice). While we implemented a strategy for lung lavage that would maximize volume returned, thereby maximizing bacterial signal from the lungs (and minimizing the likelihood of a false negative conclusion), the dilution of BAL fluid is a recognized source of variability in both humans and mice and may have impacted our results. Our study only tested the use of whole lung tissue and BAL fluid for the purposes of amplicon-based sequencing and may yield different results if other sequencing methods (e.g., metagenomic sequencing) are applied. Whole lung tissue contains much more host DNA than bacterial DNA, which can confound attempts at metagenomic analyses due to the depth of sequencing required to return reliable bacterial data [72], and may contribute to the lower numbers of reads observed in whole lung tissue in our study. Given the impossibility of performing both BAL and whole lung homogenization on the same mouse, as lavaging the lungs before the collection of whole lung tissue would confound the comparison of the two specimen types, we could not perform paired analysis on the same mice. Based on prior results [6], we assumed that co-housed mice from the same vendor and shipment should have lung bacterial communities with similar taxonomic composition, but it remains possible that mouse-to-mouse variation may have confounded some comparisons. Finally, despite our efforts to thoroughly account for all possible sources of bacterial signal found in both types of lung specimens, it is possible that we have not accounted for all potential source communities, including occult sources of contamination or other body sites in contact with the lungs, such as the nasopharynx and blood.

## Conclusions

We here present evidence supporting the use of whole lung tissue over BAL fluid in murine lung microbiome studies. The use of an ecology-based experimental and analytic approach highlights the importance of sequencing, analyzing, and reporting ample negative controls and, to the extent possible, contiguous anatomical sites or other biological source communities to assess the reality of bacterial signal in low-biomass microbiome studies.

## Supplementary Information

**Additional file 1.** Online Data Supplement

## Data Availability

The dataset supporting the conclusions of this article is available in the NIH Sequence Read Archive (accession number: PRJNA644805) at https://www.ncbi.nlm.nih.gov/bioproject/PRJNA644805/. The script used for mothur analysis can be found at https://github.com/piyuranjan/DicksonLabScripts/blob/master/mothurGreatLakes.sh. R code and accompanying files for the microbial community analysis and statistical tests presented in this paper can be found at https://github.com/dicksonlunglab/WholeLungvBALFluid.

## Abbreviations

BAL: Bronchoalveolar lavage
DNA: Deoxyribonucleic acid
OTU: Operational taxonomic unit
PCA: Principal component analysis
PCR: Polymerase chain reaction
PBS: Phosphate-buffered saline
ddPCR: Droplet digital PCR
SEM: Standard error of the mean
HSD: Honest significant difference
