# Correction to: Whole lung tissue is the preferred sampling method for amplicon-based characterization of murine lung microbiota

**DOI:** 10.1186/s40168-021-01121-x

**Published:** 2021-07-04

**Authors:** Jennifer M. Baker, Kevin J. Hinkle, Roderick A. McDonald, Christopher A. Brown, Nicole R. Falkowski, Gary B. Huffnagle, Robert P. Dickson

**Affiliations:** 1 Department of Microbiology and Immunology, University of Michigan Medical School, Ann Arbor, MI 48109 USA; 2 Department of Internal Medicine, Division of Pulmonary and Critical Care Medicine, University of Michigan Health System, 6220 MSRB III/SPC 5642, 1150 W. Medical Center Dr, Ann Arbor, MI 48109-5642 USA; 3 Department of Molecular, Cellular, & Developmental Biology, University of Michigan, Ann Arbor, MI 48109 USA; 4 Mary H. Weiser Food Allergy Center, University of Michigan Medical School, Ann Arbor, MI 48109 USA; 5 Michigan Center for Integrative Research in Critical Care, Ann Arbor, MI USA

**Correction to: Microbiome 9, 99 (2021)**

**https://doi.org/10.1186/s40168-021-01055-4**

Following the publication of the original article [1], the author reported that there were corrections not correctly carried out. On page 4, two occurences of 30,000 rpm should have read 13,000 rpm

The original article has been updated.
