# Supplementary material for: Whole lung tissue is the preferred sampling method for amplicon-based characterization of murine lung microbiota
Source: Microbiome. 2021 May 5;9:99. doi: 10.1186/s40168-021-01055-4 (PMC8101028; doi:10.1186/s40168-021-01055-4)
Supplement: Supplementary file 2 — Additional file 1. Online Data Supplement [file 40168_2021_1055_MOESM2_ESM.docx]

Online Data Supplement for “Whole lung tissue is the preferred sampling method for amplicon-based characterization of murine lung microbiota”

**Authors**: Jennifer M. Baker, Kevin J. Hinkle, Roderick A. McDonald, Christopher A. Brown, Nicole R. Falkowski, Gary B. Huffnagle, and Robert P. Dickson

**
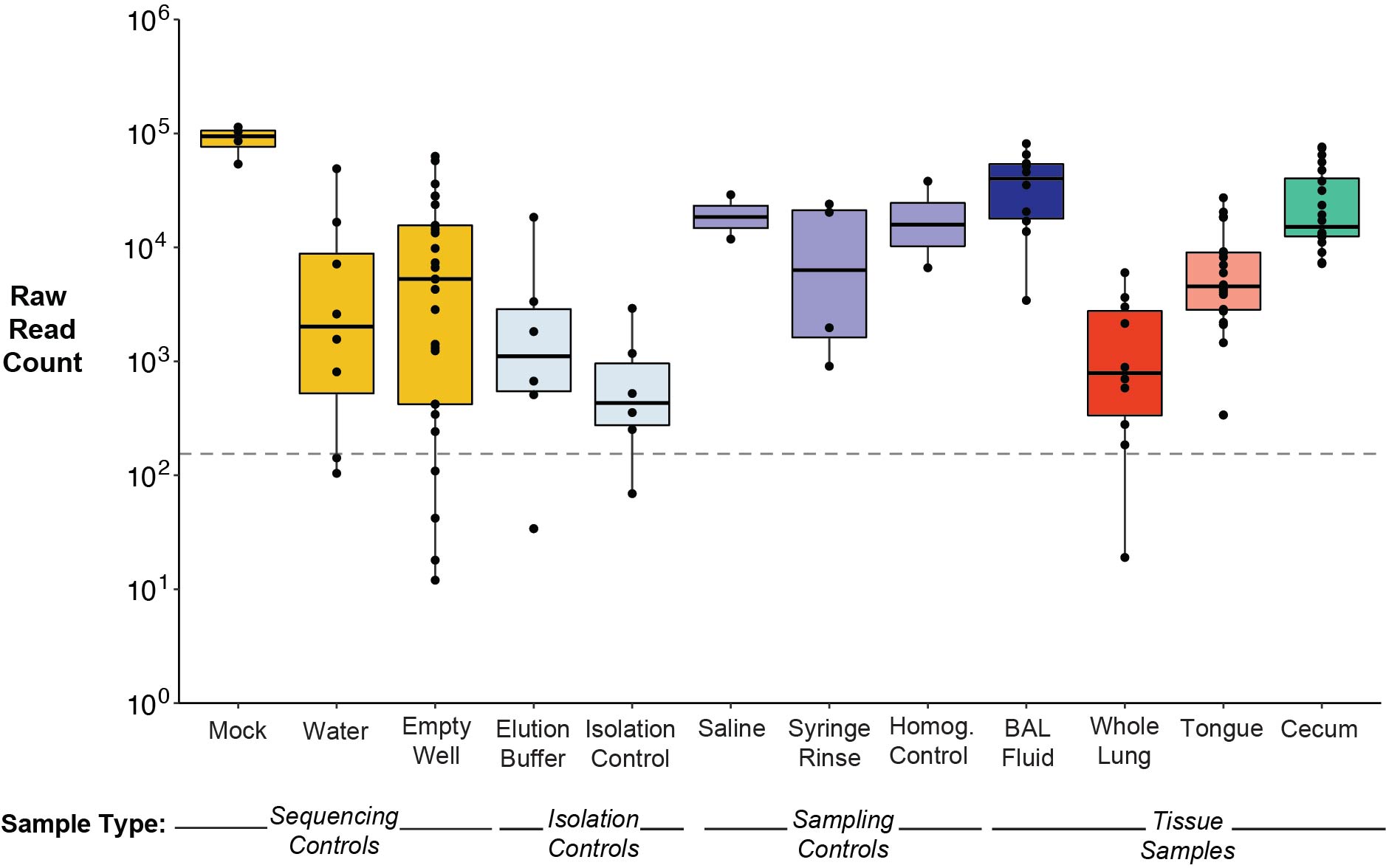
**

**Supplementary Figure 1: Quality 16S rRNA gene sequencing data was obtained for all sample types.** A. Sufficient numbers of reads were obtained for all sample types. Dotted line represents the minimum number of identified reads required to be included in further analysis (≥ 150 reads). One whole lung tissue specimen with 19 reads (shown below the dotted line) did not meet the minimum requirement for read count and was excluded from the main analysis. Eight other negative control samples returned less than 150 reads and were also excluded from the analysis. Median and IQR are shown. Individual points represent number of identified bacterial reads obtained for each tissue or control specimen post-mothur processing.

**Supplementary Figure 2: Increased alpha diversity of bacterial communities in murine whole lung tissue is driven by increased number of unique taxa relative to BAL fluid and negative controls.** Within-sample diversity of bacterial communities in whole lung tissue was comparable to that of tongue specimens and significantly greater than that of sampling, isolation, and sequencing controls. In contrast, the bacterial communities in BAL fluid were not significantly more diverse than sampling controls. Within-sample diversity of bacterial communities was quantified using the Shannon diversity index, which accounts for both richness and evenness of species diversity. When considered together with Figure 3, the difference in alpha diversity between whole lung tissue and BAL fluid appears to be driven by increased richness (number of unique OTUs) in whole lung tissue, with comparable evenness in both specimen types resulting in a non-significant difference in Shannon diversity index. Mean ± SEM and individual data points are shown. Pairwise significance was determined by comparing whole lung tissue and BAL fluid to pooled sampling, isolation, and sequencing controls (respectively, as shown) using Tukey’s HSD test. Significance key: ns p > 0.05; * p ≤ 0.05; ** p ≤ 0.01; *** p ≤ 0.001; ****p ≤ 0.0001.


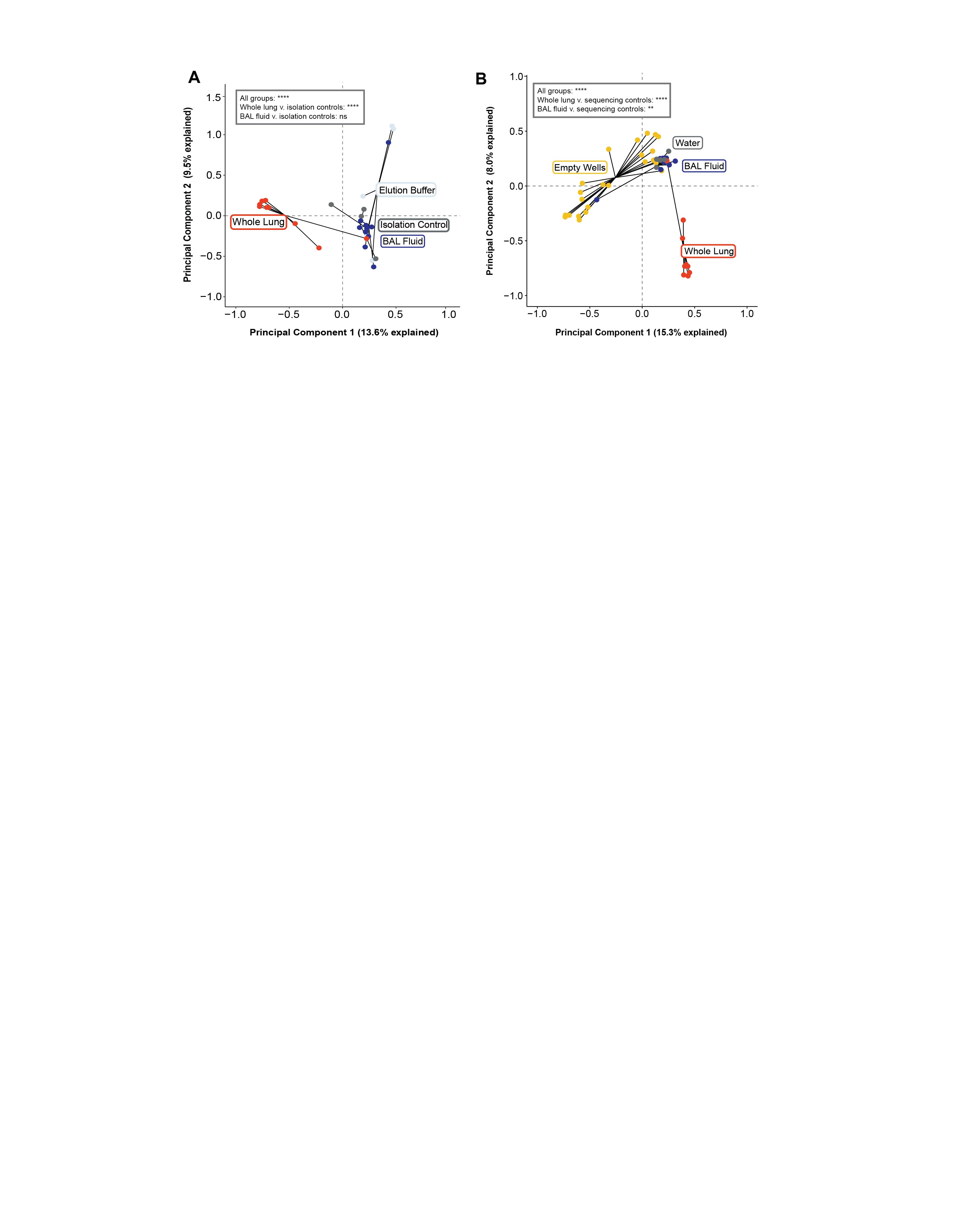


**Supplementary Figure 3: The taxonomic composition of bacterial communities in murine whole lung tissue is distinct from isolation and sequencing controls.** A. Whole lung tissue clusters separately from BAL fluid and isolation controls. B. Whole lung tissue clusters separately from BAL fluid and sequencing controls. For all panels, plots were generated by principal component analysis of Hellinger-transformed 16S rRNA gene sequencing data. Points represent individual specimens grouped by sample or control type. Overall significance was determined by permutational multivariate ANOVA. Pairwise significance was determined by two-sample PERMANOVA, conducted by pooling the isolation or sequencing controls, respectively, and comparing to each lung sample type. Significance key: ns p > 0.05; * p ≤ 0.05; ** p ≤ 0.01; *** p ≤ 0.001; ****p ≤ 0.0001.

**Supplementary Figure 4: Murine whole lung tissue displays OTU-level similarity to the oral microbiome and BAL fluid displays OTU-level similarity to negative controls.** A. Whole lung tissue displays greater overlap in OTUs present in tongue specimens than does BAL fluid. B. BAL fluid displays a greater number of OTUs present in negative controls relative to whole lung tissue. Percent relative abundance of OTUs ranked by average abundance in (A) tongue specimens or (B) pooled sampling, isolation, and sequencing negative controls. Bars represent mean abundance ± SEM of the top 50 OTUs present in (A) tongue specimens or (B) pooled negative controls, found in each sample type. Labels denote genus (or most specific taxonomic level if no genus was assigned) and unique identifier for each OTU.

**Supplementary Table 1: Summary of tissue samples and controls**

| **Sample Type** | **Tissue or Control?** | **Type of Control** | **Purpose** | **N** | **Paired?** |
| --- | --- | --- | --- | --- | --- |
| BAL Fluid | Tissue | - | Primary outcome – murine lung microbiota sampling method | 10 | No – independent variable in experiment |
| Whole Lung | Tissue | - | Primary outcome – murine lung microbiota sampling method | 10 (9) | No – independent variable in experiment |
| Tongue | Tissue | - | Paired low-biomass oropharyngeal source community (microaspiration) | 20 | Yes – one per mouse |
| Cecum | Tissue | - | Paired high-biomass control and gastrointestinal source community (coprophagy) | 20 | Yes – one per mouse |
| Zymo Mock Community | Control | Positive Sequencing Control | Sample of known microbial community composition – to detect sequencing errors | 4 | No – overall procedural control |
| Sterile Saline | Control | Negative Sampling Control for BAL Fluid | To detect background bacterial DNA in saline used for lavage | 2 | No – overall procedural control |
| Syringe Rinse | Control | Negative Sampling Control for BAL Fluid | To detect background bacterial DNA in lavage apparatus | 4 | No – overall procedural control |
| Homogenization Control | Control | Negative Sampling Control for Whole Lung | To detect background bacterial DNA in water and on tissue homogenizer used to process whole lung | 2 | No – overall procedural control |
| Isolation Control | Control | Negative Isolation Control | To detect background bacterial DNA in DNA isolation kit reagents | 6  (5) | No – overall procedural control |
| Elution Buffer | Control | Negative Isolation Control | To detect background bacterial DNA in buffer used to elute isolated DNA | 6  (5) | No – overall procedural control |
| Sterile Water | Control | Negative Sequencing Control | To detect background bacterial DNA in water used to prepare libraries | 8  (6) | No – overall procedural control |
| Empty Wells | Control | Negative Sequencing Control | To detect background bacterial DNA in 96 well plate used for library sequencing | 28  (23) | No – overall procedural control |
| No Template Control | Control | Negative ddPCR control | To detect background bacterial DNA in water and reagents used for ddPCR quantification | 4 | No – overall procedural control |

**Supplementary Table 1: Implementation of several types of tissue and reagents controls enabled the effective comparison of lung sampling approaches.** A description of each sample or control type and rationale for use in this study is outlined in the table. N indicates the number of tissue samples or controls sequenced via 16S rRNA gene amplicon sequencing (or quantified via ddPCR, in the case of the no template control), with the number in parentheses indicating the number of samples or controls retained for the main analysis after excluding samples that did not meet the minimum read requirement (see Supplementary Figure 1). If no samples were excluded, no number in parentheses is given.

**Supplementary Table 2: Comparison of hypothesis test results from raw, trimmed, and final sequencing datasets**

| **Figure** | **Statistical**  **Test** | **Comparison**  **Groups** | **Untrimmed (Raw) Dataset** | **Trimmed Dataset** | **Untrimmed & Quality-Checked Dataset** | **Trimmed & Quality-Checked**  **(Final) Dataset** |
| --- | --- | --- | --- | --- | --- | --- |
| 3 | Tukey’s HSD | whole lung v.  BAL fluid | 0.0150895 (*) | **-** | **0.0000234 (****)** | **-** |
| 3 | Tukey’s HSD | whole lung v.  sampling controls | 0.0087438 (**) | **-** | **0.0000000 (****)** | **-** |
| 3 | Tukey’s HSD | whole lung v.  isolation controls | 0.0000253 (****) | **-** | **0.0000000 (****)** | **-** |
| 3 | Tukey’s HSD | whole lung v. sequencing controls | 0.0000000 (****) | **-** | **0.0000000 (****)** | **-** |
| 3 | Tukey’s HSD | BAL fluid v.  sampling controls | 0.9958863 (ns) | **-** | **0.1880839 (ns)** | **-** |
| 3 | Tukey’s HSD | BAL fluid v.  isolation controls | 0.4262449 (ns) | **-** | **0.0018475 (**)** | **-** |
| 3 | Tukey’s HSD | BAL fluid v.  sequencing controls | 0.0757056 (ns) | **-** | **0.0000003 (****)** | **-** |
| 4 | BH-corrected pairwise Wilcoxon | whole lung v.  BAL fluid | 3.8 x 10^-12^ (****) | 1.3 x 10^-10^ (****) | **-** | **6.3 x 10^-9^ (****)** |
| 4 | BH-corrected pairwise Wilcoxon | whole lung v.  empty wells | 7.1 x 10^-7^ (****) | 5.2 x 10^-7^ (****) | **-** | **1.2 x 10^-5^ (****)** |
| 4 | BH-corrected pairwise Wilcoxon | BAL fluid v.  empty wells | 0.29581 (ns) | 0.27300 (ns) | **-** | **0.2994 (ns)** |
| 5A | Multivariate  PERMANOVA | all | 0.000099 (****) | 0.000099 (****) | **-** | **0.000099 (****)** |
| 5A | Two-sample PERMANOVA | whole lung v.  negative controls | 0.0002 (***) | 0.0002 (***) | **-** | **0.0004 (***)** |
| 5A | Two-sample PERMANOVA | whole lung v.  BAL fluid | 0.000099 (****) | 0.000099 (****) | **-** | **0.000099 (****)** |
| 5A | Two-sample PERMANOVA | BAL fluid v.  negative controls | 0.4382 (ns) | 0.464 (ns) | **-** | **0.463 (ns)** |
| 5B | Multivariate  PERMANOVA | all | 0.000099 (****) | 0.000099 (****) | **-** | **0.000099 (****)** |
| 5B | Two-sample PERMANOVA | whole lung vs.  tongue | 0.009199 (**) | 0.009199 (**) | **-** | **0.0101 (*)** |
| 5B | Two-sample PERMANOVA | BAL fluid vs.  tongue | 0.000099 (****) | 0.000099 (****) | **-** | **0.000099 (****)** |
| 5C | Mann-Whitney U test | whole lung vs.  BAL fluid | 0.0002057 (***) | 0.0002057 (***) | **-** | **0.0004114 (***)** |
| S2 | Tukey’s HSD | whole lung v.  BAL fluid | 0.3728207 (ns) | 0.3602822 (ns) | **-** | **0.0929873 (ns)** |
| S2 | Tukey’s HSD | whole lung v.  sampling controls | 0.0071118 (**) | 0.0077170 (**) | **-** | **0.0001784 (***)** |
| S2 | Tukey’s HSD | whole lung v.  isolation controls | 0.0000143 (****) | 0.0000190 (****) | **-** | **0.0000000 (****)** |
| S2 | Tukey’s HSD | whole lung v. sequencing controls | 0.0000000 (**** | 0.0000000 (****) | **-** | **0.0000000 (****)** |
| S2 | Tukey’s HSD | BAL fluid v.  sampling controls | 0.3911169 (ns) | 0.4191854 (ns) | **-** | **0.1653939 (ns)** |
| S2 | Tukey’s HSD | BAL fluid v.  isolation controls | 0.0106434 (*) | 0.0141500 (*) | **-** | **0.0000491 (****)** |
| S2 | Tukey’s HSD | BAL fluid v.  sequencing controls | 0.0000534 (****) | 0.0000800 (****) | **-** | **0.0000000 (****)** |
| S3A | Multivariate PERMANOVA | all | 0.000099 (****) | 0.000099 (****) | **-** | **0.000099 (****)** |
| S3A | Two-sample  PERMANOVA | whole lung vs. isolation controls | 0.000099 (****) | 0.000099 (****) | **-** | **0.000099 (****)** |
| S3A | Two-sample  PERMANOVA | BAL fluid vs.  isolation controls | 0.05969 (ns) | 0.07029 (ns) | **-** | **0.1785 (ns)** |
| S3B | Multivariate PERMANOVA | all | 0.000099 (****) | 0.000099 (****) | **-** | **0.000099 (****)** |
| S3B | Two-sample  PERMANOVA | whole lung vs. sequencing controls | 0.000099 (****) | 0.000099 (****) | **-** | **0.000099 (****)** |
| S3B | Two-sample  PERMANOVA | BAL fluid vs. sequencing controls | 0.0004 (***) | 0.0004 (***) | **-** | **0.0023 (**)** |

**Supplementary Table 2: Conclusions drawn from hypothesis test results were consistent across stages of quality filtering.** All values indicate the p-value from the specified comparison using the datasets indicated. The untrimmed (raw) dataset refers to the sequencing data as processed by mothur before exclusion of low-frequency OTUs and samples with insufficient reads. The trimmed dataset refers to the raw dataset after exclusion of low-frequency OTUs, but without exclusion of any samples; OTUs that comprised greater than 0.1% of reads for one or more samples were retained in the trimmed dataset. Untrimmed and quality-checked dataset was used for community richness calculation since trimming of OTUs is not appropriate when quantifying the number of bacterial taxa present in each sample. The trimmed and quality-checked (final) dataset refers to the trimmed dataset after exclusion of samples with insufficient numbers of reads. Only tissue or control samples that met the minimum number of reads were retained in the quality-checked final dataset (Supplementary Figure 1). Bolded p-values indicate comparisons included in the referenced figure.
